# Comparing individual-level clinical data from antenatal records with routine health information systems indicators for antenatal care in the West Bank: A cross-sectional study

**DOI:** 10.1371/journal.pone.0207813

**Published:** 2018-11-27

**Authors:** Mahima Venkateswaran, Kjersti Mørkrid, Khadija Abu Khader, Tamara Awwad, Ingrid K. Friberg, Buthaina Ghanem, Taghreed Hijaz, J. Frederik Frøen

**Affiliations:** 1 Global Health Cluster, Division for Health Services, Norwegian Institute of Public Health, Oslo, Norway; 2 Centre for Intervention Science in Maternal and Child Health (CISMAC), University of Bergen, Bergen, Norway; 3 Palestinian National Institute of Public Health, World Health Organization, Ramallah, Palestine; 4 Ministry of Health, Ramallah, Palestine; Ghana Health Services, GHANA

## Abstract

**Background:**

In most low- and middle-income settings, national aggregate health data is the most consistently available source for policy-making and international comparisons. In the West Bank, the paper-based health information system with manual aggregations is transitioning to an individual-level data eRegistry for maternal and child health at the point-of-care. The aim of this study was to explore beforehand how routine health information systems indicators for antenatal care can change with the introduction of the eRegistry.

**Methods:**

Data were collected from clinical antenatal paper records of pregnancy enrollments for 2015 from 17 primary healthcare clinics, selected by probability sampling from five districts in the West Bank. We used the individual-level data from clinical records to generate routinely reported health systems indicators. We weighted the data to produce population-level estimates, and compared these indicators with aggregate routine health information systems reports.

**Results:**

Antenatal anemia screening at 36 weeks was 20% according to the clinical records data, compared to 52% in the routine reports. The clinical records data showed considerably higher incidences of key maternal conditions compared to the routine reports, including fundal height discrepancy (20% vs. 0.01%); Rh-negative blood group (6.8% vs. 1.4%); anemia with hemoglobin<9.5 g/dl (6% vs. 0.6%); and malpresentation at term (1.3% vs. 0.03%). Only about a sixth of cases with these conditions were referred according to guidelines to designated referral clinics.

**Conclusions:**

Differences between indicators from the clinical records data and routine health information systems reports can be attributed to human error, inconsistent denominators, and complexities of data processes. Key health systems indicators were prone to underestimations since their registration was dependent on referral of pregnant women. With a transition to individual-level data, as in the eRegistry under implementation, the public health authorities will be able to generate reliable health systems indicators reflective of the population’s health status.

## 2 Introduction

The monitoring of global progress in reproductive, maternal, newborn and child health hinges on the routine availability of good quality data [1–3]. Low and middle-income countries (LMIC) typically rely on common sources of data for decision-making, such as censuses and population-based surveys, and to a lesser extent on clinical records and other forms of provider-reported data [4, 5]. The majority of process indicators to assess the delivery of essential interventions in maternal and child health are not amenable to measurement solely through population-based surveys [6, 7]. Strengthening of routine data collections in health facilities is important, since these data may be the most suitable source for many maternal and child health indicators [8–10]. Globally, there has been a sustained call for improving the quality and availability of data from Routine Health Information Systems (RHIS) [11–14]. Despite this, RHIS data for maternal and child health are often lacking in most LMIC settings and if available are incorrect, incomplete or of poor quality [8, 15–17]. There are increasing efforts to improve health system-wide data collection in many LMIC with electronic health information systems, although most of these systems focus on collection of aggregated data [18]. Data aggregation, however, is fraught with its own issues, such as incorrect and inconsistent definitions of the indicators and denominators and errors in counting, and this is partly due to RHIS reporting processes and partly due to behavioral factors [19–23]. The indicators collected in RHIS seemingly have little direct consequence on delivery of health services and it is sometimes challenging to impart the importance of good quality routine data collection to the care providers [24–27].

The paper-based health information system at the primary healthcare level in the West Bank is now transitioning to an eRegistry for maternal and child health [25, 28, 29]. The eRegistry will in the future compute and automatically generate RHIS indicators from individual-level clinical data collected by care providers at the point-of-care in primary healthcare clinics, thus eliminating the need for manual aggregations and reporting [25].

The objective of this study was to compute routinely reported indicators from individual-level clinical data from antenatal paper records, mimicking an eRegistry, and compare these with indicators reported in the existing health information system in the West Bank.

## 3 Materials and methods

**Fig 1:**
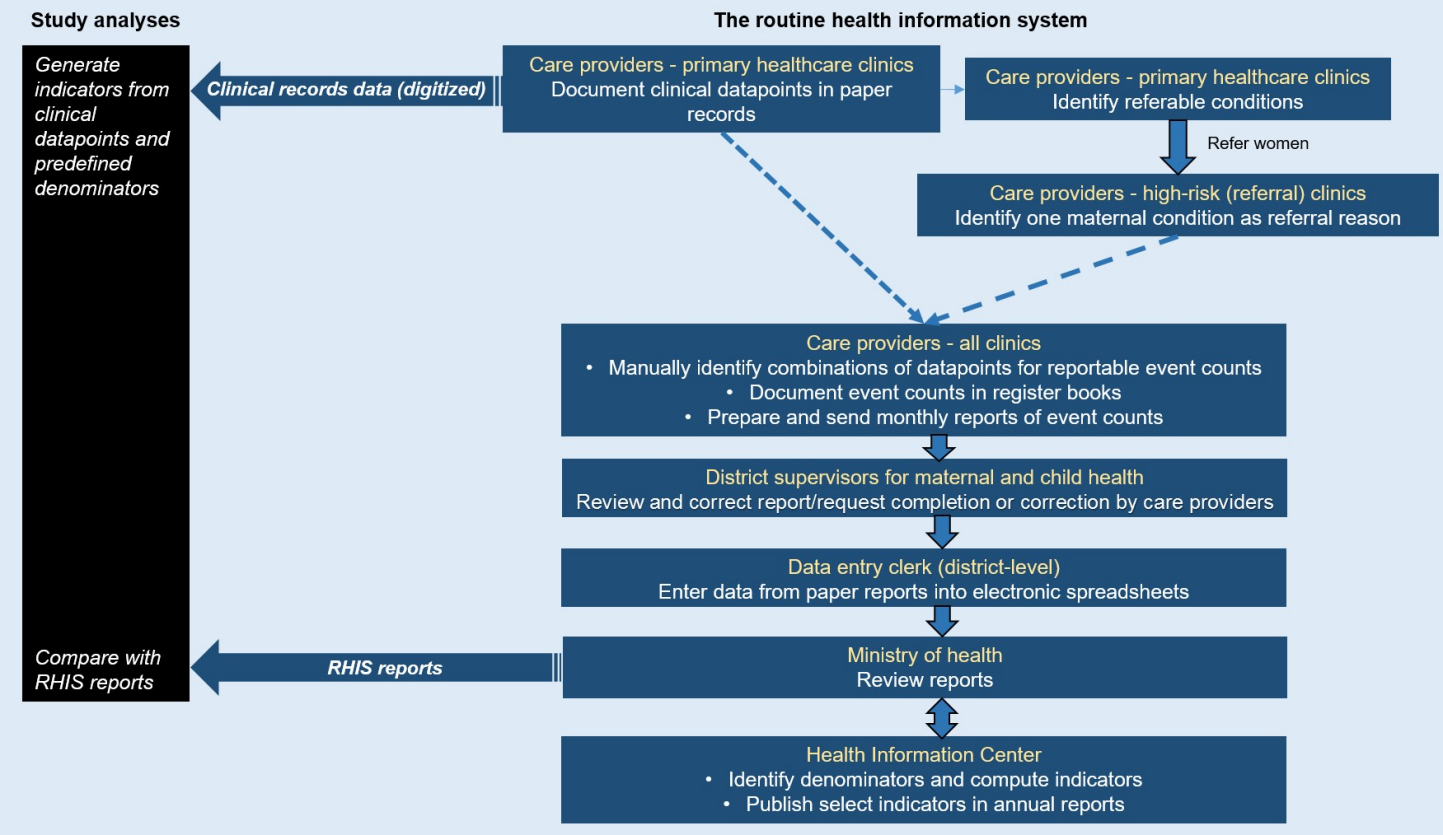
Aggregate reporting in the paper-based routine health information in the West Bank, Palestine, and sources of data used for analyses in this study.

First, we selected indicators of antenatal care that were routinely reported in the health system in the West Bank. We used data from clinical records available from a cross-sectional sample of primary healthcare clinics to generate the selected indicators (Fig 1). These indicators were then compared with the indicator values in aggregate RHIS reports available at the clinic-, district- and national levels (Fig 1).

### 3.1 Study setting

In the West Bank, two types of healthcare facilities provide antenatal care in the public health system–primary healthcare clinics and referral clinics, also known as high-risk clinics [28]. According to the clinical guidelines in the public health system, when pregnant women are detected with certain conditions during antenatal care in the primary healthcare clinics, they are referred to pre-specified high-risk clinics [28].

In the paper-based RHIS in primary healthcare in the West Bank, care providers first document data in clinical records in the primary healthcare clinics. The clinical records used for antenatal care consist of socio-demographic data; obstetric, surgical and medical history; lab test results and ultrasound examinations. Using the clinical records, care providers manually identify and aggregate reportable conditions and events, and document the event counts in dedicated register books on a daily or weekly basis. Aggregate monthly reports of event counts are then prepared and sent from all primary healthcare clinics to the district-level, and subsequently to the national health authorities (Fig 1). The high-risk clinics, in addition, report on the number of maternal conditions observed in referred women who attend care, and submit aggregate reports on behalf of all primary healthcare clinics from which they receive referrals in each district (Fig 1). A select list of RHIS indicators are published annually as part of national health reports for the West Bank with statistics reported per district [30]. In the West Bank, obstetric services are only provided at the hospitals, but they do not report to the RHIS on maternal conditions that may have been identified during antenatal care.

In 2016, the first phase of the national implementation of the eRegistry was launched with the intention to include five districts in the West Bank [28, 29]. In preparation for the eRegistry implementation, data, equivalent to the planned data in the eRegistry, was extracted from paper-based clinical records for the year of 2015 in a random sample (see below) of primary healthcare clinics. According to an inventory assessment of the primary healthcare clinics in Palestine completed in 2014, these clinics enrolled about 11,400 pregnancies a year, an average of 70 pregnancies per clinic per year [28, 29]. These clinics referred to one of 9 high-risk clinics located in the five districts [28].

### 3.2 Sample size and sampling

Sample size estimations for data collection from the clinical records were made using ‘OpenEpi’ for a population size of 11,400, aiming to enable the detection of a frequency of 1% for the least prevalent outcome in the population (for example, severe anemia in pregnancy) with an absolute precision of 0.5% [31]. A minimum sample of 1344 clinical records was required, corresponding to all pregnancies registered over a year from 15–20 clinics.

Primary healthcare clinics were selected using the probability proportional to size method, in order to obtain a data set of pregnancies that was representative of the healthcare received by pregnant women in the West Bank [32]. Selection was continued until a minimum number of clinics was available to achieve the required sample size, provided the clinical records of all pregnant women enrolled over one year in the sampled clinics were included in the data collection.

### 3.3 Indicators

To enable the comparisons we selected antenatal care indicators that were routinely reported to the RHIS by the health system, and could be computed in an identical manner with data from clinical records (Table 1). We then ascertained the definitions, diagnostic classifications and data categorizations of the indicators as they are intended to be used for aggregate RHIS reporting. We excluded from our analyses those indicators that cannot be computed using datapoints from clinical records, such as antenatal supplementation of iron and folic acid that was reported in the RHIS as number of units prescribed. Conditions such as preterm rupture of membranes and antepartum vaginal bleeding were part of RHIS reporting, but were excluded since women with these conditions were most likely referred to hospitals and these data were, therefore, unlikely to be accurately collected in clinical records.

**Table 1:** Routinely reported indicators of antenatal care in the RHIS selected for analysis–definitions and data needs for computation from clinical records data.

Serial number	RHIS indicator included in analyses	Definition for computation of event counts (numerators)	Datapoints from clinical records for computations
1.	Antenatal visits (mean)	Total number of antenatal visits, total number of pregnancies enrolled	
2.	Maternal age	Age of woman at the time of registration of pregnancy*	Date of birth of the pregnant woman; date of first antenatal visit
3.	Anemia: maternal anemia at 36 weeks	Pregnant women who have Hb less than 11 g/dl at 35–38 gestational weeks	Lab test: Hb (g/dl); gestational age^ᵻ^
4.	Reportable maternal conditions from referrals		
4.1	Gestational diabetes mellitus	Women with a random blood sugar > = 140 g/dl or a 1 hour 50 g oral glucose challenge test of > = 140 mg/dl	Lab test: random blood sugar, oral glucose challenge test
4.2	Multiple pregnancy	Women with multiple pregnancy	Ultrasound examination: number of fetuses
4.3	Malpresentation at term	Non-cephalic presentations at or after 36 gestational weeks	Ultrasound examination: fetal presentation; gestational age^ᵻ^
4.4	Recurrent miscarriage	Three consecutive pregnancy losses prior to 20 gestational weeks	Obstetric history: 3 or more consecutive pregnancy losses prior to 20 gestational weeks
4.5	Preeclampsia^1^ [33]	New onset hypertension plus new onset proteinuria after 20 weeks of gestation; hypertension defined as a systolic blood pressure of 140 mm Hg or greater, and/or a diastolic blood pressure of 90 mm Hg or greater	Clinical examination: systolic and diastolic blood pressures (mm Hg); lab test: proteinuria; gestational age^ᵻ^
4.6	History of Cesarean sections	Cesarean section(s) in the previous delivery(ies)	Obstetric history: previous delivery/ies by Cesarean section
4.7	Anemia: at any gestational age	Pregnant women who ever have a Hb<9.5 g/dl	Lab test: Hb (g/dl); gestational age^ᵻ^
4.8	Rhesus negative blood group	Pregnant women with a Rhesus negative blood group	Lab test: Rhesus typing of blood group
4.9	Fundal height discrepancy	A symphysis fundus height measurement of more or less than 2 cm compared to gestational age (in weeks) at the time of measurement	Clinical examination: symphysis fundus height values; gestational age^ᵻ^
4.10	Oligohydramnios or polyhydramnios	Pregnant women with an ultrasound-detected increase or decrease in amniotic fluid	Ultrasound examination: diagnosis of oligohydramnios or polyhydramnios**

RHIS: Routine Health Information System; Hb: Hemoglobin

^1^American College of Obstetricians and Gynecologists. Task Force of Hypertension in Pregnancy.

^ᵻ^ Best estimate of gestational age computed from the dates of visits/ lab tests and date of last menstrual period, or from ultrasound estimated expected date of delivery.

*Categorized as <16 and >40 years according to the reporting requirement in the RHIS.

**No defined diagnostic criteria, subject to clinical diagnosis.

### 3.4 Data extraction

#### 3.4.1 Clinical records data

Two trained nurse-midwives completed the data extraction during January–April 2017, and entered data from paper-based clinical records into electronic data entry forms hosted on the District Health Information System 2 (DHIS2) software platform [34]. Data from approximately 10% of all antenatal records were entered by both the data extractors, and these data were checked for quality and consistency [28, 29].

#### 3.4.2 Aggregate RHIS reports

For the comparisons, RHIS reports of aggregate event counts and indicators were obtained from the Ministry of Health as electronic spreadsheets (Table 2). Event counts for three of the selected indicators were available in the RHIS reports sent from the primary healthcare clinics (RHIS clinic reports) (Table 2). Event counts of reportable maternal conditions were available at the district-level, and reported from the high-risk (referral) clinics (RHIS district reports) (Table 2). All the indicators were part of the publicly available RHIS national reports (nationally reported statistics for the five districts) (Table 2).

**Table 2:** Data sources used for comparative analyses and their descriptions. *

Name of data source used in the study	Generated from	Sample for analyses and comparison	Data content	Indicators available/generated	Denominator used for computing indicators in the study
Clinical records data	Primary healthcare clinics	Clinical paper records from probability sample of 17 clinics, cross-sectional data	Clinical datapoints	All	All pregnant women registered for antenatal care from 17 primary healthcare clinics, whose clinical records were extracted (n = 1369)
RHIS clinic reports	Primary healthcare clinics	Aggregate RHIS reports from 17 clinics	Event counts	Maternal age, antenatal visits, anemia at 36 weeks	Number of pregnancies enrolled as reported by care providers (n = 1463)
RHIS district reports	High-risk (referral) clinics	Aggregate RHIS reports from 9 high-risk clinics	Event counts	Maternal conditions from referrals	Pregnancies enrolled in clinics that refer to the high-risk clinics in the study area^¥^ (n = 11,416)
Nationally reported statistics	Health Annual Report [30]	Aggregate RHIS reports of national statistics**	Event counts; proportion indicators ^ᵻ ᵻ^	All	Pregnancies enrolled in clinics in study- and non-study areas (n = 14,544)

RHIS: Routine Health Information System; Hb: Hemoglobin

*all data and indicators are for the year 2015 for 5 districts in the West Bank, Palestine.

^¥^ refers to the area in the five districts from where the sample for this study was derived.

**contains values of all event counts/indicators sent from primary healthcare clinics and high-risk (referral) clinics as part of the RHIS.

^ᵻ ᵻ ^anemia at 36 weeks published as a percentage of total hemoglobin tests with value <11g/dl, of all hemoglobin tests reported.

### 3.5 Analyses

#### 3.5.1 Clinical records data

We used the clinical datapoints and definitions of indicators listed in Table 1 to reconstitute each of the selected indicators from the clinical records data. Gestational ages were computed from the first day of the woman’s last menstrual period, according to usual clinical practice in this context. If these data were missing, the ultrasound estimated expected date of delivery was used to calculate gestational ages. We calculated prevalence of reportable maternal conditions in the entire sample as well as the occurrence of maternal conditions only among referred women. The latter was similar to how these indicators were generated as part of the aggregate RHIS reporting. Only test or examination results were documented in the paper records, and “no data” in these data fields were interpreted as a test or examination not performed.

Sample weights were added such that pregnant women from smaller clinics were assigned higher weights than those from larger clinics (the inverse of the probability of the clinic being selected, as to create data that can be compared to the RHIS district reports) [35]. Analyses were carried out using STATA version 15 (StataCorp. 2017. Stata Statistical Software: Release 15. College Station, TX: StataCorp LLC), and the STATA command svyset was used to calculate weighted proportions and 95% confidence intervals (CI) [36].

#### 3.5.2 Aggregate RHIS reports

Event counts from the aggregate RHIS reports were transformed to proportions with 95% confidence intervals using pre-defined denominators (Table 2).

### 3.6 Ethics approval

Ethical clearance was obtained from the Palestinian Health Research Council (PHRC/HC/272/17) and the Regional Committee for Medical and Health Research Ethics in Norway (2017/1537-3). We adhered to the Palestinian Ministry of Health’s legal framework in obtaining access to anonymized data for secondary analyses [29].

## 4 Results

Seventeen primary healthcare clinics from 5 districts in the West Bank were included in the data collection and data from clinical records were available for 1369 pregnancies enrolled for antenatal care in 2015 in these clinics. Of these, 501 women (37%) were nulliparous. Sixteen-per-cent (n = 222) of the women were <20 years of age and 9% (n = 118) were >35 years age at the time of enrollment at the clinic. Complete RHIS clinic reports for 2015 were obtained from all the primary healthcare clinics that were included in the data collection of the clinical records (n = 17) (Table 2). RHIS district reports were available from all their corresponding high-risk clinics (n = 9) (Table 2).

### 4.1 Maternal age

There was consistency in the indicator maternal age at pregnancy registration between the clinical records data (age<16 years: 0.1%, 95% CI: 0–0.4; age>40 years: 1.2%, 95% CI: 0.6–2.1), RHIS clinic reports (age<16 years: 0.1%, 95% CI: 0–0.4; age>40 years: 1.4%, 95% CI: 1–2), and the nationally reported statistics for the five districts (age<16 years: 0.2%, 95% CI: 0.1–0.3; age>40 years: 1.7%, 95% CI: 1.5–2).

### 4.2 Antenatal visits

The number of antenatal visits per pregnant woman in the clinical records data (mean = 4.5; standard deviation = 2.3), RHIS clinic reports (mean = 4.5) and nationally reported statistics for the five districts (mean = 4.7) were all comparable.

### 4.3 Anemia at 36 weeks

The proportion of women with anemia at 36 weeks in the clinical records data (32%, 95% CI: 22–44) was similar to the RHIS clinic reports (31%, 95% CI: 29–35) and the nationally reported statistics for the five districts (30%, 95% CI: 29–31). However, there were 280 documented hemoglobin tests at 36 weeks in the clinical records data, representing a 20% anemia screening coverage at 36 weeks, compared to 890 reports of such hemoglobin tests (61% screening coverage) in the RHIS clinic reports. According to the nationally reported statistics for the five districts, there were 7602 hemoglobin tests at 36 weeks (52% screening coverage).

### 4.4 Reportable maternal conditions

In the clinical records data, the incidences of malpresentation at term (1.3%; 95% CI: 0.6–2.8), anemia (hemoglobin<9.5 g/dl) (6%, 95% CI: 4.1–8.7), Rh-negative blood group (6.8% 95% CI: 4.5–10.2) and fundal height discrepancy (20%; 95% CI: 12.4–30.8) were higher compared to the incidence of these reportable conditions for referral in the RHIS district reports and nationally reported statistics for the five districts (Table 3). In the clinical records data, 7% (95% CI: 6–9) of the women had two and 1% (95% CI: 0.5–2) had three of the reportable maternal conditions.

**Table 3:** Routinely reported maternal conditions from antenatal care–comparison of indicators from all clinical records data and only referred women, and aggregate RHIS reports.

Reportable condition	Clinical records data—all* (N = 1369)		Clinical records data—occurrence of condition and referred**	RHIS district reports (N = 11,416)		RHIS national statistics (N = 14,544)	
	n	Weighted % (95% CI)	Weighted % (95% CI)	n	% (95% CI)	n	% (95% CI)
Gestational diabetes mellitus	12	0.8 (0.4–1.7)	0.05 (0.01–0.4)	79	0.7 (0.6–0.9)	79	0.5 (0.4–0.7)
Multi-fetal pregnancy	20	1.3 (0.8–2.0)	0.4 (0.2–1.0)	84	0.7 (0.6–0.9)	97	0.7 (0.5–0.8)
Malpresentation at term	20	1.3 (0.6–2.8)	0.2 (0.1–0.7)	2	0.02 (0–0.06)	4	0.03 (0.01–0.07)
Recurrent miscarriages	26	1.7 (0.9–3.5)	0.7 (0.2–2.4)	144	1.3 (1.1–1.5)	150	1.0 (0.2–3.0)
Preeclampsia	7	0.6 (0.2–1.3)	0.2 (0.02–1.2)	26	0.2 (0.1–0.3)	31	0.2 (0.1–0.3)
History of Cesarean sections	93	6.4 (4.1–9.7)	2.2 (1.3–3.6)	631	5.5 (5.1–5.9)	777	5.3 (4.9–5.7)
Anemia (Hb<9.5 g/dl)	88	6.0 (4.1–8.7)	0.9 (0.4–2.0)	87	0.8 (0.6–0.9)	93	0.6 (0.5–0.8)
Rh-negative blood group	95	6.8 (4.5–10.2)	1.2 (0.6–2.1)	180	1.6 (1.4–1.8)	202	1.4 (1.2–1.5)
Fundal height discrepancy	253	20 (12.4–30.8)	0.9 (0.5–1.6)	None	None	1	0.01 (0–0.04)

RHIS: Routine Health Information Systems; CI- confidence interval; Hb- hemoglobin

*No cases of oligohydramnios or polyhydramnios in the clinical data

**Estimates of indicators after accounting for missed data in the RHIS reporting from women not being referred according to guidelines

**Fig 2:**
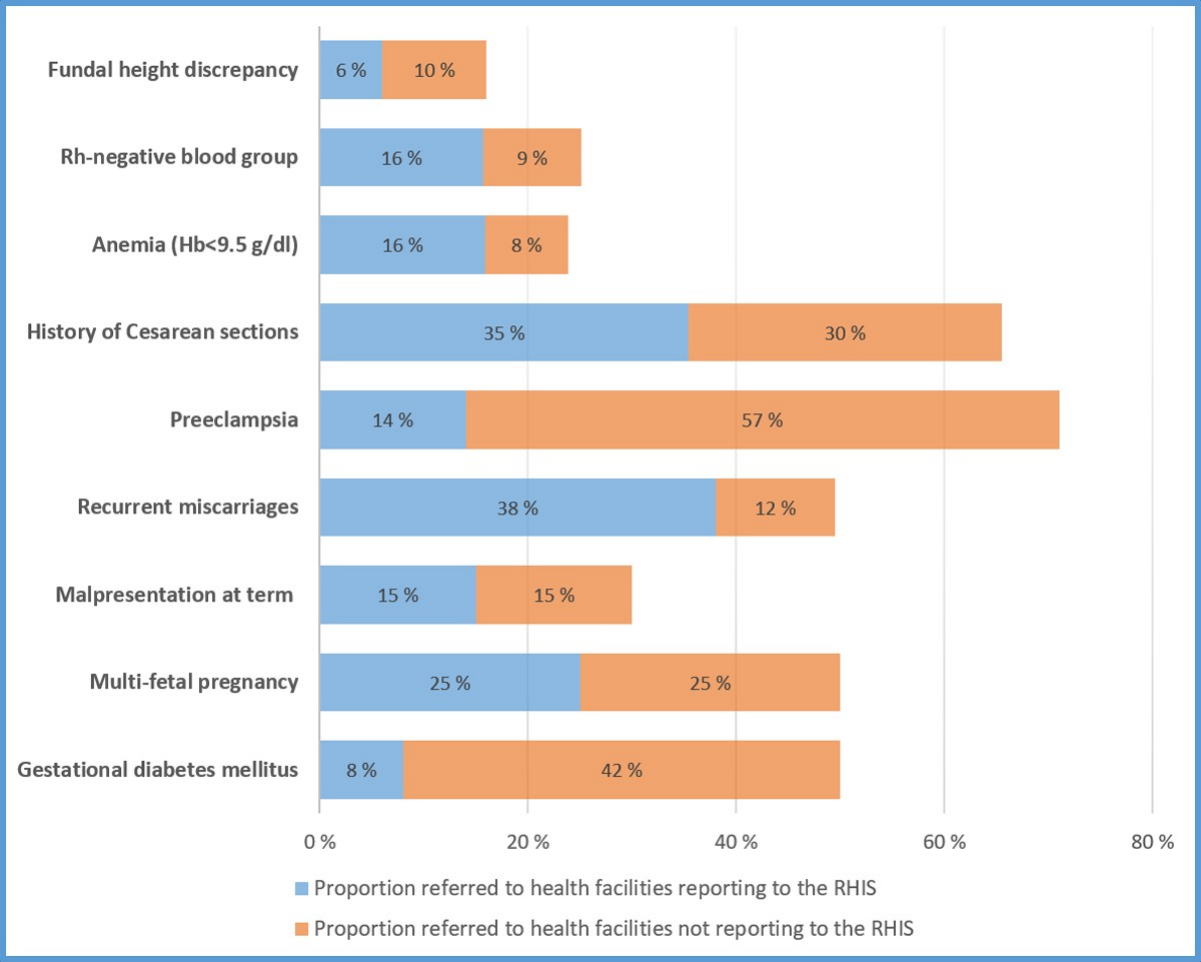
Women with maternal conditions that were referred to health facilities that report to the RHIS, and to health facilities that do not report on antenatal care indicators to the RHIS. RHIS: Routine health information system.

According to the clinical records data, the proportion of women with a documented referral from the primary healthcare clinics to any health facility, ranged from 16% for fundal height discrepancy to 71% for preeclampsia (Fig 2). Proportions that were referred to the pre-specified high-risk clinic for reportable maternal conditions were lower (Fig 2).

If reportable maternal conditions were estimated only among pregnant women that were referred to high-risk clinics, malpresentation at term (0.2%, 95% CI: 0.1–0.7) and fundal height discrepancy (0.9%, 95% CI: 0.05–1.7) were the only two conditions that continued to have a higher value in the clinical records data compared to RHIS reports (Table 3).

For all routinely reported aggregate RHIS indicators of antenatal care, there was consistency between what was reported by the primary healthcare clinics (RHIS clinic reports) and high-risk clinics (RHIS district reports), and the publicly available national reports (Table 3).

## 5 Discussion

Appraisal of RHIS data and indicators are important components of assessment of health systems [37, 38]. In this study, we compared RHIS reports with individual-level data from clinical records, which revealed important pitfalls in the generation of the indicators, and these would have been missed by only performing consistency checks of reports within the existing RHIS. The divergences between the clinical records data and RHIS reports were due to previously recognized issues with RHIS in general, such as inconsistent denominators for calculating indicators, errors in manual computations, and production of unreliable indicators due to a complex reporting structure in the health system [17, 26, 39].

RHIS reporting of maternal anemia at 36 weeks was an illustration of an indicator with an inconsistent denominator. Apart from reporting an overall higher number of hemoglobin tests at 36 weeks compared to the clinical records data, three out of the 17 primary healthcare clinics reported more hemoglobin tests than the total number of pregnancies enrolled in 2015, and appeared to be including hemoglobin tests of pregnancies enrolled in the previous year. With the denominator reported and used in the RHIS for maternal anemia, it was neither feasible to estimate the true incidence of maternal anemia for a given year of reporting nor quantify the coverage of hemoglobin testing.

In our study, issues with manual computations were particularly evident for the indicator fundal height discrepancy. The gestational ages documented by the care providers often varied from the gestational ages generated for this study. While some care providers may have determined fundal height discrepancy based on the current exact gestational age, others may have used the nearest completed gestational week. For example, a gestational age of 30 weeks and three days may be interpreted as 30 weeks or 31 weeks. Using the gestational ages documented by the care providers for computing this indicator from the data in the clinical records yielded an incidence of fundal height discrepancy of 9% (95% CI: 4–19), which was still higher than the RHIS reports (0.01%, 95% CI: 0–0.04). Additional reasons for the observed difference between the clinical records data and RHIS reports for this indicator include the lack of more comprehensive fetal growth monitoring strategies, non-compliance to guidelines to refer women with any fundal height discrepancy as per the existing definition, and known issues in the measurement itself [40–42]. Ultrasound examinations during antenatal care are reportedly widely used in the West Bank. Given this, ultrasound-based fetal growth monitoring may take precedence over serial fundal height measures. However, there were neither diagnostic standards nor reporting guidelines for results from other forms of screening of fetal growth.

Three factors relating to a complex RHIS reporting process contributed to the disparity in the reportable maternal conditions between the clinical records data and RHIS reports. First, maternal morbidities (except maternal anemia at 36 weeks) were reported from the high-risk clinics and not from the referring primary healthcare clinics, making the registration of the indicators conditional on referral and utilization of care. However, there was low compliance of the primary healthcare clinics to the recommended guidelines for referrals to high-risk clinics (Fig 1). Second, in the RHIS district reports, only one reportable maternal condition was registered per referred pregnant woman. Third, there were notable variations among the districts in the selection of the principal maternal condition for reporting to the RHIS. In one of the 5 districts, history of Caesarean sections constituted 26% of all the reported maternal conditions and gestational diabetes mellitus 9%. In another district, 55% of all the reporting was for history of Cesarean sections, with gestational diabetes mellitus constituting less than 1%.

Similarly, there may be variations in referral practices among the primary healthcare clinics. In the clinical records data from the sample of clinics included in this study, a lower proportion of women with history of Cesarean sections (2.2% vs. 5.3%) and gestational diabetes mellitus (0.05% vs. 0.5%) were referred to the high-risk clinics, compared to RHIS reports.

Other studies in the West Bank have reported Cesarean section rates of at least 14–23% [30, 43]. The proportions of women with history of Cesarean sections from the clinical records data as well as RHIS reports were clear underestimations, probably due to incomplete documentation of this datapoint in the clinical records.

The generalizability of all RHIS indicators can be improved by adopting more standardized definitions. As an illustration, if the World Health Organization’s diagnostic cut-off for fasting blood sugar levels was used for computations of the clinical records data, the resulting incidence of gestational diabetes mellitus was 6% (95% CI: 4–10), compared to the 0.8% (95% CI: 0.4–1.7) obtained from the clinical records data using the current definition in the public health system [44].

The reporting of the mean number of antenatal visits is not representative of antenatal care coverage for an individual. The variability in antenatal visits for individual pregnant women was evident from the wide standard deviation (SD = 2.3) around the mean.

A strength of this study was its ability to identify issues beyond the quality of RHIS data and processes, such as variations in adherence to guidelines for referrals as well as selective reporting of indicators in the health system. The quality of healthcare services may be improved by understanding and addressing issues related to referrals. The public health authorities may need to revisit the value of certain guidelines for referral, particularly for non-critical conditions during pregnancy. The feasibility and effectiveness of different fetal growth monitoring strategies in primary healthcare for this population are themes for future research. One of the functionalities of the eRegistry, the interactive checklists and clinical decision support, provides guideline-based recommendations for referral and clinical reminders for the care providers at the point-of-care in the primary healthcare clinics [28].

One limitation of this study is that only data that were documented in the antenatal records were considered in the analyses. We have regarded any undocumented visits or tests as not having occurred. Some primary healthcare clinics may have additional or alternative sources of documentation that are used specifically for the purpose of RHIS reporting, particularly for lab test results (for example, for reporting of maternal anemia at 36 weeks). Lack of exclusive use of clinical records for all documentation by the care providers may also explain the differences in the number of new enrollments of pregnancies from the clinical records data (n = 1369) and RHIS reports (n = 1463). About 50% of all pregnant women in the West Bank receive antenatal care in the private and non-governmental sector that are not part of RHIS reporting for antenatal care, and the incidences of maternal conditions reported in this study may not be representative of the entire population of the West Bank.

## 6 Conclusion

The eRegistry for maternal and child health aims to eliminate sources of errors that impact the quality of health systems data, by using individual-level clinical data to directly produce RHIS reports at the individual, clinic, sub-national and national levels. As the health system in the West Bank shifts from manually aggregated data to the eRegistry, it will be possible to generate more reliable and complete health systems indicators.
